# Transcriptional Pattern Analysis of Virus-Specific CD8+ T Cells in Hepatitis C Infection: Increased Expression of TOX and Eomesodermin During and After Persistent Antigen Recognition

**DOI:** 10.3389/fimmu.2022.886646

**Published:** 2022-06-06

**Authors:** Nils H. Wildner, Andreas Walker, Franziska Brauneck, Vanessa Ditt, Sven Peine, Samuel Huber, Friedrich Haag, Claudia Beisel, Joerg Timm, Julian Schulze zur Wiesch

**Affiliations:** ^1^ I. Department of Medicine, Section of Infectious Diseases, University Medical Center Hamburg-Eppendorf, Hamburg, Germany; ^2^ Institute of Virology, Medical Faculty, University Hospital Düsseldorf, Heinrich-Heine-Universität, Düsseldorf, Germany; ^3^ II. Department of Medicine, Center for Oncology, University Medical Center Hamburg-Eppendorf, Hamburg, Germany; ^4^ Department of Transfusion Medicine, University Medical Center Hamburg-Eppendorf, Hamburg, Germany; ^5^ German Center for Infection Research Deutsches Zentrum für Infektionsforschung (DZIF), Partner Site Hamburg-Lübeck-Borstel-Riems, Hamburg, Germany; ^6^ Institute of Immunology, Center for Diagnostics, University Medical Center Hamburg-Eppendorf, Hamburg, Germany

**Keywords:** chronic viral hepatitis, hepatitis C, acute viral hepatitis, T cell differentiation, T cell exhaustion, TOX, Eomesodermin, CD39

## Abstract

Thymocyte selection-associated high mobility group box (TOX) has been described to be a key regulator in the formation of CD8+ T cell exhaustion. Hepatitis C virus (HCV) infection with different lengths of antigen exposure in acute, chronic, and after resolution of HCV infection is the ideal immunological model to study the expression of TOX in HCV-specific CD8+ T cells with different exposure to antigen. HCV-specific CD8+ T cells from 35 HLA-A*01:01, HLA-A*02:01, and HLA-A*24:02 positive patients were analyzed with a 16-color FACS-panel evaluating the surface expression of lineage markers (CD3, CD8), ectoenzymes (CD39, CD73), markers of differentiation (CD45RO, CCR7, CD127), and markers of exhaustion and activation (TIGIT, PD-1, KLRG1, CD226) and transcription factors (TOX, Eomesodermin, T-bet). Here, we defined on-target T cells as T cells against epitopes without escape mutations and off-target T cells as those against a “historical” antigen mutated in the autologous sequence. TOX+HCV-specific CD8+ T cells from patients with chronic HCV and on-target T cells displayed co-expression of Eomesodermin and were associated with the formation of terminally exhausted CD127^-^PD1^hi^, CD39^hi^, CD73^low^ CD8+ T cells. In contrast, TOX+HCV-specific CD8+ T cells in patients with off-target T cells represented a progenitor memory Tex phenotype characterized by CD127^hi^ expression and a CD39^low^ and CD73^hi^ phenotype. TOX+HCV-specified CD8+ T cells in patients with a sustained virologic response were characterized by a memory phenotype (CD127+, CD73^hi^) and co-expression of immune checkpoints and Eomesodermin, indicating a key structure in priming of HCV-specific CD8+ T cells in the chronic stage, which persisted as a residual after therapy. Overall, the occurrence of TOX+HCV-specific CD8+ T cells was revealed at each disease stage, which impacted the development of progenitor Tex, intermediate Tex, and terminally exhausted T cell through an individual molecular footprint. In sum, TOX is induced early during acute infection but is modulated by changes in viral sequence and antigen recognition. In the case of antigen persistence, the interaction with Eomesodermin leads to the formation of terminally exhausted virus-specific CD8+ T cells, and there was a direct correlation of the co-expression of TOX and Eomes and terminally exhausted phenotype of virus-specific CD8+ T cells.

## Introduction

Spontaneous resolution of acute hepatitis C virus (HCV) infection is characterized by a broad, strong, and long-lasting virus-specific CD8+ and CD4+ T cell response (1, 2). On the other hand, a dysfunctional immune response is a critical factor in the outcome and pathogenesis of the disease (3, 4). The chronic stage of HCV infections is characterized by a nearly absent virus-specific CD4+ T cell response and a functionally deficient CD8+ T cell response with an additional viral escape to this CD8+ T cell response, leading to poor clinical outcomes (5–8). Furthermore, the persistence of the T cell immune exhaustion pattern was demonstrated even after therapy (9–11).

In murine and human chronic viral infections, virus-specific CD8+ T cells highly express multiple inhibitory receptors including PD-1, TIGIT, 2B4, and CD39, and show weak functionality in a state which has been termed T cell exhaustion (7, 12–16). T cell exhaustion is a distinct form of T cell differentiation, within which progenitor and terminally differentiated subsets are distinguished (17–19). The pathway of differentiation seems to be governed by a complex orchestra of expression of transcription factors by which differentiation between progenitor, intermediate, and terminal Tex cells is achieved (20–23). In this context, Tex cell progenitor cells are defined by the expression of CXCR5 or TCF1 (24, 25). Tex cells are capable of proliferating in response to PD-1 blockade (7, 25). Intermediate Tex T cells are characterized by T-bet expression and downregulation of TCF-1 (19, 25). These cells are used as precursors to Eomes^hi^ T cells which still seem to have effector potential (19, 20, 26). In terminally exhausted T cells, on the other hand, an increase in the transcription factor Eomesodermin (Eomes) has been observed (19, 20, 27). Although different transcription factors are involved at different stages of differentiation, TOX was revealed to be present in both precursors and terminally exhausted T cells (28–30). Interestingly, depletion of TOX results in a reduction of PD-1 expression, stronger cytotoxic functionality, and a reduction of Tex progenitor cells (28). However, a reduced survival capacity of TOX-depleted T cells has also been observed (28).

Especially in the chronic stage of HCV infection, terminally exhausted (TCF-1^-^CD127^-^PD1^hi^) CD8+ T cells upregulate Eomes and show impaired cytokine production whereas the transcriptional factor TCF1 is upregulated in fully functional, virus-specific memory (CD127^+^PD1^+^) CD8+ T cells (7, 31–33).

Besides the detection of TOX in the LCMV mouse model, upregulation of TOX has also been demonstrated in human viral infectious diseases such as chronic hepatitis B (HBV) (34), human immunodeficiency virus (35), HCV (11, 28), Epstein-Barr virus (34, 35), cytomegalovirus (34, 35), and influenza (FLU) (11, 28).

To understand to what extent findings from murine experiments concerning T cell exhaustion in the LCMV model translate to humans, hepatitis C infection is the ideal model that can provide an understanding of TOX expression and cooperation with other transcription factors and correlation with immune checkpoint molecules, activation, and differentiation markers during acute, chronic, and post-resolution of viral infection and different levels of antigen exposure (36, 37).

In addition to the identification of possible immune checkpoint molecules involved in T cell exhaustion, metabolic pathways are also of additional interest for the development of further immunomodulatory therapies. The ectonucleotidase CD39 together with CD73 can convert extracellular proinflammatory adenosine triphosphate (ATP) into immunomodulatory adenosine (ADO) (38). This signaling cascade as a metabolic regulator of the immune system is of increasing interest in the pathogenesis of T cell exhaustion in infectious diseases and cancer. CD39 has even been termed a potential immune checkpoint molecule that could be an important therapeutic tool in combinational immune therapy (39–41). It has already been described that the decrease of CD73+ T cells during infection with, for example, HIV is associated with immune activation (42). Also, in malignant diseases, for example, AML and melanoma, a disturbance of the microenvironment (hypoxic and low ADO) could be shown (43, 44). Besides their role as immunomodulatory enzymes, CD39 and CD73 are also involved in T cell differentiation. Thus, an increase in CD39 and a decrease in CD73 are observed during T cell activation and the formation of effector T cells (42). Further understanding of the regulation and function of these two ectoenzymes in exhausted T cells could help to develop a new level personalized therapy (42, 45, 46).

Limited information is available on the expression pattern of TOX at different phases of HCV infection, including acute infection and the reversibility of the exhaustion phenotype after viral resolution after therapy or HCV- specific T cells that are specific to epitopes with evidence of mutation of the circulating virus (28, 34). Both detailed analyses of the phenotype of TOX+ virus-specific CD8+ T cells and analysis of the transcriptional profile and differentiation, activation, and exhaustion phenotype of HCV-specific T cells at different stages of the disease with HCV are needed. Here, the molecular network of HCV virus-specific CD8+ T cells was analyzed using tetramer enrichment technology to provide a better insight into the role of TOX in the pathogenesis of HCV infection.

To further explore the cellular and transcriptional signatures and programs, we used 16 color flow cytometry analyses and detected the level of differentiation, the co-expression of activation, and inhibitory receptors in parallel with the related transcription factors.

## Material And Methods

### Patient Cohort and PBMC Isolation

Cryopreserved peripheral blood mononuclear cells (PBMC) of hepatitis C infected patients (n=35) at different phases of HCV infection, all collected at the University Medical Center Hamburg–Eppendorf, were used for immunophenotypic staining. A written agreement was given by all patients. This study was approved by the local ethics board of the Ärztekammer Hamburg WF14-09, PV4780, PV4081. PBMCs from these patients were used to perform MHC-class I tetramer and multicolor flow cytometry analysis.

### HLA Typing

HLA typing was performed at the Institute of Transfusion Medicine at the University Medical Center Hamburg-Eppendorf. For this, they used the PCR-SSO commercial kit SSO LabType (One Lambda, Canoga Park, CA).

### MHC Class I Tetramer Staining and Enrichment

We used different MHC class I tetramers to detect the virus-specific CD8+ T cells in this study. MS columns were used for a magnetic bead associated with the MHC class I tetramer enrichment technique as previously described (12). For this PBMCs were thawed and stained with PE- or APC-labeled HLA matching tetramers and enriched by using MACS technology according to the manufacturer’s protocol. We first stained the HCV samples with the PE-labeled HLA matching tetramers using the HCV panel (**Supplementary Figure S2**). Then, the FLU enrichment was performed with the APC-labeled HLA matching tetramers with the depleted fraction of the samples using the FLU panel (**Supplementary Figure S2**). Tetramer fractions were further analyzed by flow cytometry using BD LSRFortessa™.

### Multiparametric Flow Cytometry

Cells were stained for multiparametric flow cytometry after the tetramer enrichment as previously described (12). To exclude dead cells, PBMC were stained with the LIVE/DEAD™ Fixable Near-IR dye (Thermo Fisher, Germany) according to the manufacturer´s protocol. PBMC were surface stained with fluorochrome coupled surface antibodies, including BUV395 CD226 (Clone: J168-540, Catalog-number: 742498), BUV737 CD39 (Clone: TU66, Catalog-number: 612852), BV421 TIGIT (Clone: A15153G, Catalog-number: 372710), BV510 CD45RO (Clone: UCHL1, Catalog-number: 304246), BV605 CD73 (Clone: AD2, Catalog-number: 344024), BV650 CCR7 (Clone: G043H7, Catalog-number: 353233), BV785 CD127 (Clone: A019D5, Catalog-number: 351330), FITC KLRG1 (Clone: MAFA, Catalog-number: 367714), PerCp-Cy5.5 CD8 (Clone: Rpa-T8, Catalog-number: 301031), PE-Cy7 PD1 (Clone: EH12.2H7, Catalog-number: 329918), and Alexa Fluor 700 CD3 (Clone: OKT3, Catalog-number: 317341). PBMCs were permeabilized for 45 min for intracellular staining using the Bioscience™ Foxp3/Transcription Factor Staining Buffer Set according to the manufacturer´s protocol. PBMC were intracellularly stained with fluorochrome coupled intracellular antibodies, including BV711 T-bet (Clone: 4B10, Catalog-number: 644819), PE TOX (Clone: REA473, Catalog-number: 130-120-785), PE-eFluor610 Eomesodermin (Clone: WD1928, Catalog-number: 61-4877-42), and APC TOX (Clone: REA473, Catalog-number: 130-118-335) for 45 min in the refrigerator at 4°C in the dark. BUV395 anti-CD226 and BUV737 anti-CD39 were purchased from BD Bioscience (Heidelberg, Germany), PE-eFluor 610 anti-Eomesodermin was purchased from eBioscience (Thermo Fisher, Germany), and all other antibodies were purchased from BioLegend (Koblenz, Germany). The gating strategy is shown in **Supplementary Figure S1** and the panels used are shown in their entirety in **Supplementary Figure S2**.

### Sequencing of Patient Isolates

Sequencing of HCV was done as previously described (47, 48). In brief, viral RNA was extracted with the QIAamp viral RNA kit (Qiagen, Hilden, Germany) according to the manufacturer’s protocol. RNA was transcribed with Superscript III (Invitrogen) with the reverse primer Oligo d(A) (49). HCV amplicons were amplified in a two-step nested PCR using GoTaq Polymerase (Promega) and genotype-specific primers (**Supplementary Table S1**). PCR conditions were: 120 s at 94°C followed by 35 cycles each 30 s 94°C, 30 s 55°C, and 160 s 72°C followed by 10 min at 72°C. The PCR products were directly sequenced, and sequences were aligned with the software Geneious 10.2.6 (Biomatters, Auckland, New Zealand).

### Statistical Analysis

The flow cytometric generated data were analyzed using FlowJo version 10.4.2 software (Treestar, Ashland, OR). Statistical analysis was carried out using Prism 6.0 software (GraphPad Software, San Diego, CA). All results were checked for normal distribution using the D’Agostino-Pearson omnibus normality test or Shapiro–Wilk normality test. For the comparison of individual nonpaired samples, we used a parametric t-test or Mann–Whitney test. For paired analyses, we used a paired t-test or Wilcoxon matched-pairs signed-rank test. For correlation analyses, a Pearson correlation for parametric and Spearman correlation for nonparametric data were performed. P-values smaller than 0.05 were considered significant, where *, **, ***, and **** indicate p-values between 0.01 and 0.05, 0.001 0.0001 and 0.001, and <0.0001, respectively. Data are expressed as means with standard deviation, respectively (as indicated in the figure legend). t-distributed Stochastic Neighbor Embedding (tSNE) analyses were performed, using the tSNE plugin in FlowJo 10.6.2 (50), analyzed marker: TOX, Eomesodermin, T-bet, PD-1, TIGIT, KLRG1, CD127, CD39, CD73, CD226, CD45RO, CCR7. FlowSOM clustering and visualization technique was used for further analysis of tSNE data; analyzed markers: TOX, Eomesodermin, T-bet, PD-1, TIGIT, KLRG1, CD127, CD39, CD73, CD226 (51). For the generation of the radar plots in **Figure 6** the RStudio 2021.09.2 + 382 on Windows was used. The following R packages were used for analysis and visualization: radarchart and chartJSRadar.

## Results

### Clinical Characteristics of the Study Cohort

In this study, we analyzed 35 patients with hepatitis C infection with different clinical stages including patients with acute hepatitis C (aHCV, n=8), chronic, treatment naïve hepatitis C (cHCV, n=19), spontaneously resolved hepatitis C (rHCV. n=7), and successfully treated patients with a sustained virologic response (SVR, n=7) (2, 12, 52). Of the 35 HCV patients, the virus-specific CD8+ T cell response was determined in 25 patients by FACS staining. Samples from six patients were analyzed at various time points in different stages of disease (**Table 1**). The clinical patient characteristics, laboratory (AST, ALT), virologic (viral load, genotype), and immunological (HLA type) parameters are shown in **Table 1** and **Supplementary Figures S3A–C**.

**Table 1:** Clinical, virological, and immunological patient characteristics.

Patient	HLA-A Type	chosen HLA-A Type	Age	Sex	AST [U/I]	ALT [U/I]	Viral load [IU/ml]	Genotype	Treatment	Outcome
aHCV1*^1^	01:01, 03:01	01:01	52	m	59	177	15100	3	/	Sp. R
aHCV2	01:01, 23:01	01:01	54	w	394	1084	30000000	3a	/	Sp. R
aHCV3	02:01, 03:01	02:01	41	m	698	1353	10000000	4	/	cHCV
aHCV4	01:01, 24:02	24:02	32	m	1039	1701	10000	n.m.	/	Sp. R
aHCV5	02:01, 24:02	24:02	31	m	1315	3158	4590000	3	/	/
aHCV6	02:01, 03:01	02:01	44	m	88	120	n.m.	1a	peg IFN + Ribavarin	cHCV
cHCV1	02:01, 03:01	02:01	69	w	n.m.	n.m.	44000	n.m.	Ledipasvir, Sofosbuvir	/
cHCV2	01:01, 30:01	01:01	37	m	n.m.	n.m.	17800000	1b	Sofusbuvir, Velpatasvir	SVR
cHCV3	01:01, 02:01	02:01	62	m	19	29	3540000	1b	Elbasvir, Grazoprevit	SVR
cHCV4	02:01, -	02:01	48	m	56	131	1580000	1b	Elbasvir, Grazoprevit	SVR
cHCV5	02:01, 03:01	02:01	71	w	n.m.	n.m.	62901	1b	Elbasvir, Grazoprevit	SVR
cHCV6*^2^	01:01, 02:01	02:01	60	m	33	71	10100000	1a	Sofusbuvir, Velpatasvir	SVR
cHCV7*^3^	02:01, 11:01	02:01	76	w	133	194	97000	1b	/	/
cHCV8*^4^	02:01, 03:01	02:01	65	w	40	51	5530000	1b	Sofusbuvir, Velpatasvir	SVR
cHCV9	02:01, -	02:01	54	w	92	126	26100000	3	Sofusbuvir, Velpatasvir	SVR
cHCV10*^5^	01:01, 03:01	01:01	28	m	51	109	284000	n.m.	/	/
cHCV11	02:01, 30:01	02:01	56	m	37	41	13500000	5	Glecaprevir, Pibrentasvir	SVR
cHCV12	01:01, 02:01	01:01	39	m	128	398	3380000	n.m.	Elbasvir, Grazoprevit	SVR
cHCV13*^6^	03:01, 24:02	24:02	71	m	37	39	10000000	1b	Glecaprevir/Pibrentasvir	SVR
rHCV1*^1^	01:01, 03:01	01:01	53	m	n.m.	n.m.	n.d.	n.m.	/	/
rHCV2	02:01, 03:01	02:01	26	m	43	51	n.d.	n.m.	/	/
rHCV3	02:01, 32:01	02:01	36	m	502	815	n.d.	1a	peg IFN + Ribavarin	/
rHCV4	01:01, 02:05	01:01	36	m	66	43	n.d.	n.m.	/	/
rHCV5	01:01, 02:01	02:01	48	w	20	20	n.d.	n.m.	/	/
SVR1	01:01,02:01	02:01	33	w	125	420	n.d.	1a	Sofusbuvir, Velpatasvir	SVR
SVR2*^2^	01:01, 02:01	02:01	61	m	n.m.	n.m.	n.d.	1a	Sofusbuvir, Velpatasvir	SVR
SVR3*^3^	02:01, 11:01	02:01	76	w	n.m.	n.m.	n.d.	1b	Paritaprevir/r, Ombiasvir/Dasabuvir	SVR
SVR4*^4^	02:01, 03:01	02:01	66	w	n.m.	n.m.	n.d.	1b	Sofusbuvir, Velpatasvir	SVR
SVR5*^5^	01:01, 03:01	01:01	29	m	26	28	n.d.	n.m.	Sofusbuvir, Velpatasvir	SVR
SVR6*^6^	03:01, 24:02	24:02	73	m	18	17	n.d.	1b	Glecaprevir/Pibrentasvir	SVR
SVR7	02:01, 03:01	02:01	69	w	n.m.	n.m.	n.d.	n.m.	Sofusbuvir, Velpatasvir	SVR

m, man; w, woman; AST, aspartate transaminase; ALT, alanine transaminase; n.m., not measured; n.d., not detected; Sp. R., spontaneous recovery; SVR, sustained virological response; *, indicates the same patients at different time points and the additional numbers 1-6 indicate which samples correspond to each other.

### Dichotomous Distribution of TOX+ HCV-Specific CD8+ T Cells in Chronic Hepatitis C Depends on On-Target T Cells and Off-Target T Cells in Relation to Autologous Circulating Virus

**Figure 1:**
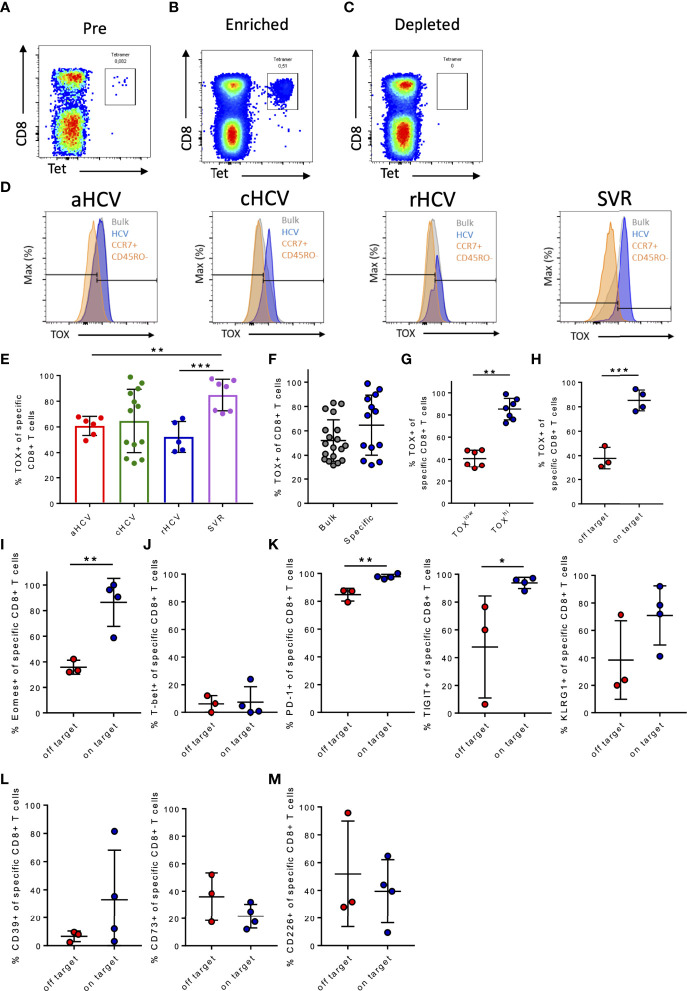
**(A–M)** Frequency of TOX+ virus-specific CD8+ T cells in chronic hepatitis C infection and comparison to the immunological phenotype of patients with on-target T cells and off-target T cells to the corresponding targeted antigen. **(A–C)** Representative dot plots of PBMC gated on CD3+ T cells before (Pre), after (Enriched), and without enrichment (Depleted). **(D)** Representative gating of marker TOX in acute HCV (aHCV), chronic HCV (cHCV), spontaneously resolved hepatitis C (rHCV), and successfully treated patients with a sustained virologic response (SVR) (blue: HCV-specific CD8+ T cells; gray: CD8+ T cells in the bulk population; orange: CCR7+ CD45RO- CD8+ T cells). **(E)** Frequencies of TOX+ virus-specific CD8+ T cells at the different stages of HCV infection. **(F)** Frequencies of TOX+ CD8+ T cells in cHCV in the bulk population and in HCV-specific CD8+ T cells. **(G)** Frequencies of TOX+ virus-specific CD8+ T cells divided into patients with low and high frequencies of TOX+ HCV-specific CD8+ T cells. **(H)** Frequencies of TOX+ virus-specific CD8+ T cells in chronic hepatitis C. **(I)** Frequencies of Eomes+ virus-specific CD8+ T cells in chronic hepatitis C. **(J)** Frequencies of T-bet+ virus-specific CD8+ T cells in chronic hepatitis C. **(K–M)** Frequency of PD-1+, TIGIT+, KLRG1+, CD39+, CD73+, and CD226+ virus-specific CD8+ T cells in chronic hepatitis C. Data are shown as mean ± SD, where *, **, and *** indicate P values <0.05, <0.01, and <0.001.

At first, to confirm the results of previous preliminary studies of the TOX expression in exhausted CD8+ T cells, the frequency of TOX+ tetramer+ HCV-specific CD8+ T cells of patients at the different stages of HCV infection was assessed (10, 11, 29). To increase the frequency of virus-specific CD8+ T cells, a tetramer-based enrichment was performed (**Figures 1A–C** **)** (12). Depending on the HLA type of the patients, a *01:01, *02:01, or a *24:02 specific tetramer was used for the analyses (**Table 2**). **Figure 1D** shows representative histogram plots for the gating of TOX+ expression (gray: bulk CD8+ T cells, blue: HCV-specific CD8+ T cells, orange: CCR7+, CD45RO- CD8+ T cells) in patients with acute, chronic, resolved hepatitis C virus infection and after treatment-induced resolution of the infection with a sustained viral response (SVR). A comparison of the TOX expression at different disease stages showed a significantly higher frequency of TOX+ HCV-specific CD8+ T cells of patients with SVR than in the acute stage of infection and with rHCV (**Figure 1E**). Surprisingly, in the chronic stage, there was a seemingly dichotomous distribution of TOX+ HCV-specific CD8+ T cells (**Figure 1E**). An increase in the frequency of TOX+ CD8+ T cells in chronic HCV infection was detected in the tetramer+ HCV-specific CD8+ T cells compared with the bulk population (**Figure 1F**). However, this difference did not reach statistical significance (p=0,0961). Here, we found a certain dichotomy of tetramer+ CD8+ T cells that were TOX^low^ and TOX^hi^ (**Figure 1G**).

**Table 2:** HLA multimeric complex information.

MHC Class I Tetramer Information			
HLA-A Molecule	HCV Protein	Position	Sequence
A*01:01	NS3	aa 1436-1444	ATDALMTGY
A*02:01	NS3	aa 1073-1081	CINGVCWTV
A*02:01	NS3	aa 1406-1415	KLVALGINAV
A*24:02	E2
HLA-A Molecule	**FLU Protein**		**Sequence**
A*01:01	Influenza NP		CTELKLSDY
A*02:01	Influenza M1		GILGFVTL

Kasprowicz et al. and others were previously able to show that changes of the autologous viral sequence occur in HCV infection and that these changes affect the exhaustion phenotype of the virus-specific CD8+ T cells (5, 8, 53). To verify whether the dichotomous distribution of TOX+HCV-specific CD8+ T cells in chronic HCV infection was related to persistent autologous sequence (on-target) or altered autologous sequence due to mutations (off-target) (54), we performed an analysis of the viral genome using bulk sequencing to identify sustained recognition of endogenous viral epitopes and HCV-specific CD8+ T cells that do not recognize endogenous viral epitopes due to the presence of viral escape mutations (8). **Table 3** shows the mutational analysis of patients with chronic hepatitis C and a positive HCV-specific CD8+ T cell response. Here, we detected three patients (cHCV4, cHCV8, cHCV10; **Table 3**) with viral escape mutations of their respective circulating viral sequence in the epitopes of interest, while four patients still had the autologous sequence (cHCV3, cHCV7, cHCV13, cHCV14). For the remaining six patients, we were unfortunately not able to perform a viral sequence analysis as we did not have corresponding plasma sample material from these patients. Patients with detected off-target T cells for the corresponding epitopes had lower frequencies of TOX+ virus-specific CD8+ T cells (**Figure 1H**).

**Table 3:** Analysis of the circulating viral genome.

Sample	Genotype	HLA-A Type	Sequence		Isolated Sequence	
cHCV3	1b	01:01	ATDALMTGY		- - - - - - - - -	
cHCV4	1b	02:01	KLVALGINAV	CINGVCWTV	- - S - - -L - - -	- - - -A - - - -
cHCV7	1a	02:01	KLVALGINAV	CINGVCWTV	- - - - - - - - - -	- - - - - - - - -
cHCV8	1b	02:01	KLVALGINAV	CINGVCWTV	- - - S - - V - - -	- - - - A - - - -
cHCV10	3a	02:01	KLVALGINAV	CINGVCWTV	- - RGM - L - - -	TVG - - M - - -
cHCV13	1b	01:01	ATDALMTGY		- - - - - - - - -	
cHCV14	1b	24:02	EYVLLLFLL		- - - - - - - - -	

(-) refers to a sequence position matching the viral wild-type sequence, capital letters represent amino acid substitutions that differ from the wild-type sequence.

We observed a significantly increased frequency of Eomes+ HCV-specific CD8+ T cells in on-target T cells to the corresponding viral sequence (**Figure 1I**). Already in some first landmark studies, a coherence of TOX and Eomes in the formation of T cell exhaustion could be described (29, 55). TOX+ and Eomes+ virus-specific CD8+ T cell frequencies in patients with cHCV were positively correlated (**Supplementary Figure S4**). In on-target as well as in the off-target tetramer+ HCV-specific CD8+ T cells, the frequency of T-bet+ HCV-specific CD8+ T cells was very low (**Figure 1J**).

Since TOX has previously been described as a transcription factor that is intricately involved in T cell exhaustion, we analyzed whether the expression of established T cell exhaustion markers differed between on-target HCV-specific CD8+ T cells as well as off-target HCV-specific CD8+ T cells (29).

For this, we examined the frequency of the expression of different immune checkpoint molecules (PD1, TIGIT, KLRG1) on HCV-specific tetramer+ CD8+ T cells of the two patient groups. Patients with on-target T cells for the corresponding sequence in the region of the HCV CD8+ tetramers showed slightly higher frequencies of the immune checkpoint molecules PD-1, TIGIT, and KLRG1 on these HCV-specific CD8+ T cells compared to the tetramer+ HCV-specific CD8+ T cells of patients with off-target T cells (**Figure 1K** **)**. We observed a trend toward lower CD39+ expression (p = 0,2286) and higher CD73+ (p = 0,2067) expression of those HCV-specific CD8+ T cells of the patients with off-target T cells albeit the difference was not significant due to the low patient numbers (**Figure 1L**). In addition, a slightly increased frequency of CD226+ virus-specific CD8+ T cells in the off-target T cells was observed (**Figure 1M** **).**

These results highlight that the transcriptional factor TOX shows a similar pattern to commonly established markers in the context of T cell exhaustion and is associated with persistent antigen stimulation (27, 29, 33).

### Multidimensional Analysis Revealed a Phenotypical Pattern of TOX+ and Eomes+ Virus-Specific CD8+ T Cells in Patients With cHCV and On-Target T Cells to the Corresponding Viral Sequence

**Figure 2:**
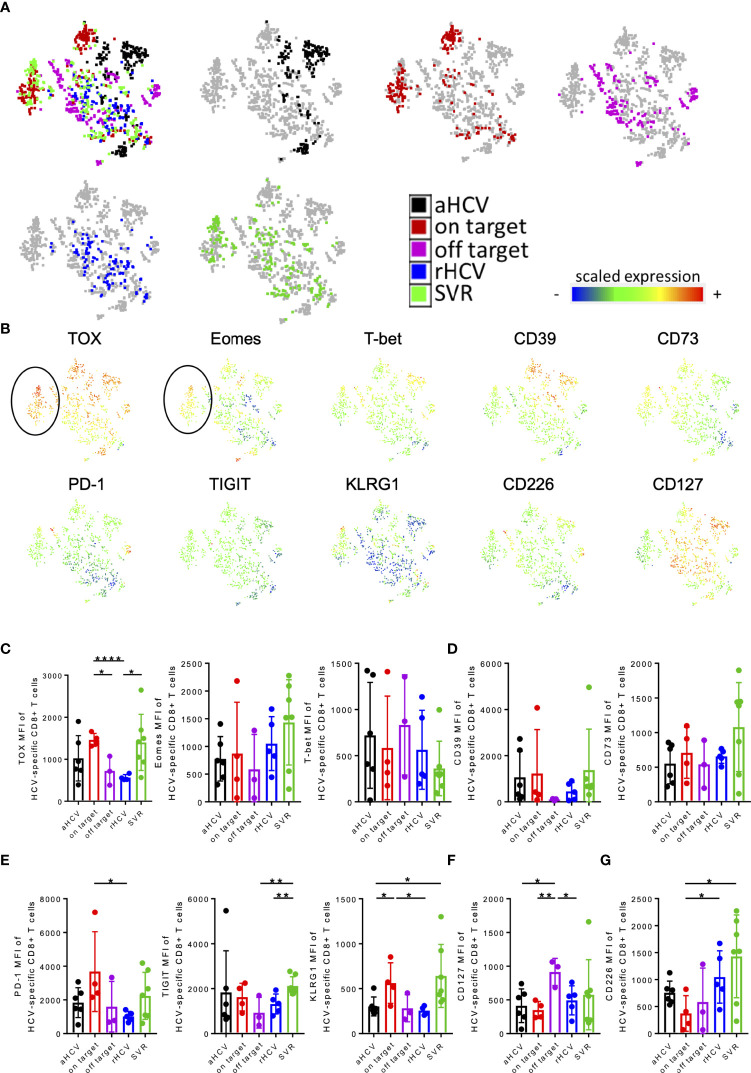
**(A–F)** tSNE and MFI analysis of virus-specific CD8+ T cells of patients aHCV, cHCV (on-target T cells and off-target T cells), rHCV, and SVR. **(A)** A 2-dimensional map of virus-specific CD8+ T cells concatenated from all selected samples was generated by t-SNE analysis. Samples from aHCV (black), cHCV (on-target; red), cHCV (off-target; purple), rHCV (blue), and SVR (green) patients were projected onto the total sample map. **(B)** The expression levels (measured as mean fluorescence intensities) of the indicated markers were projected onto the t-SNE map. **(C–G)** MFI levels of the indicated markers. Data are shown as mean ± SD, where *, **, and **** indicate P values <0.05, <0.01, and <0.0001.

To further analyze the multidimensional data of our multi-parametric analysis, and to look for a potential molecular pattern of virus-specific CD8+ T cells at different disease stages using unbiased approaches, we performed tSNE analysis looking at patients with acute HCV infection [aHCV (black)]; cHCV infection and on-target T cells [on-target (red)]; cHCV infection and off-target T cells [off-target (purple)]; resolved infection [rHCV (blue)] and successfully treated patients [SVR (green)] (**Figure 2A**) (50). We performed a FlowSOM cluster analysis of the indicated markers (**Supplementary Figure S5A**) (51). We were able to detect a cluster formation of the cHCV patients with on-target T cells and SVR patients (black circle, cluster 14) (**Figure 2B**; **Supplementary Figure S5A**). Cluster 14 represents a population of HCV-specific CD8+ T cells in patients in cHCV with on-target T cells and SVR patients characterized by high expression of the transcription factors TOX and Eomes (**Figure 2B**). In addition, the immune checkpoint molecules PD-1, TIGIT, and KLRG1 also showed a higher expression in cHCV patients with on-target T cells and patients in the stage of SVR (**Figure 2B**). On the other hand, the expression of CD226 as a marker for T cell activation and less exhausted T cells was more highly expressed in the cohort of patients who spontaneously cleared the virus and in patients with cHCV and off-target mutation (**Figure 2B**).

To validate these observations, we also analyzed and compared the MFI levels of these markers. Here, we were able to detect a significant increase in the TOX expression of the HCV-specific T cells of chronic patients with on-target T cells to the corresponding viral sequence and patients with SVR compared to patients with cHCV with mutations in the targeted epitope and rHCV (**Figure 2C**). However, regarding purinergic signaling, CD39 expression was increased in patients with cHCV and on-target viral sequence and SVR, whereas the level of CD73 expression only increased mainly on tetramer+ HCV-specific CD8+ T cells of patients with SVR (**Figure 2D**). Consistent with the tSNE data, higher MFI levels of the immune checkpoints PD-1, TIGIT, and KLRG1 were observed on HCV-specific CD8+ T cells of patients with chronic HCV and on-target viral sequence and patients with SVR (**Figure 2E**). Patients with cHCV and off-target T cells for the corresponding antigen showed the highest MFI levels for CD127 expression (**Figure 2F**). The expression of CD226 was significantly lower in cHCV patients with corresponding targeted antigen compared with the expression in patients SVR and rHCV (**Figure 2G**).

### TOX+ HCV-Specific CD8+ T Cells Exhibit a Different Molecular Pattern Depending on the Disease Stage of the HCV Infection

Recently published studies revealed that the transcription factor TOX is expressed to some extent in every Tex subset and the differentiation of Tex progenitor T cells depends on the appearance of other transcriptionfactors (e.g., TCF1, Eomes, T-bet) (28, 29, 56–58). To distinguish whether TOX+ HCV-specific CD8+ T cells differ between different HCV disease stages, next, we also compared co-expression of selected activation, differentiation, and exhaustion markers of TOX+ virus-specific CD8+ T cells.

**Figure 3:**
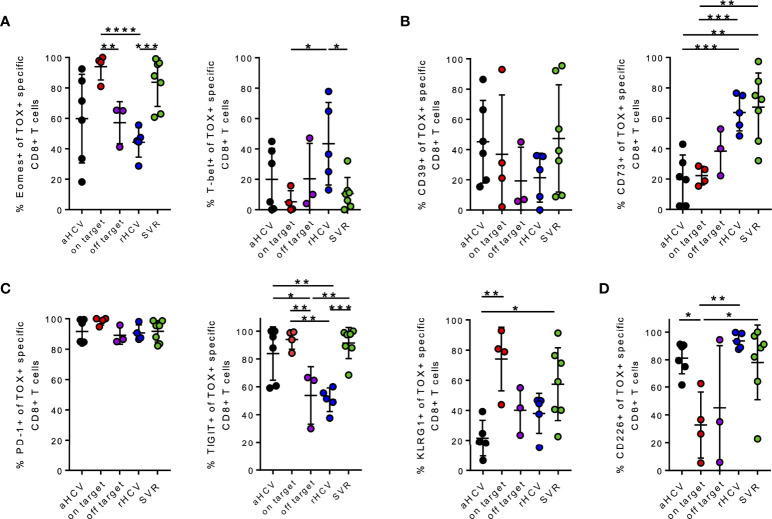
**(A–D)** Phenotypic analysis of the indicated markers on TOX+ virus-specific CD8+ T cells at different stages of hepatitis C infection. **(A–D)** Co-expression of the indicated markers of TOX+ virus-specific CD8+ T cells in all different stages of hepatitis C infection; acute HCV = aHCV, chronic HCV with on-target T cells = on target, chronic HCV with off-target T cells = off target, spontaneously resolved HCV =rHCV, and successfully treated patients with a sustained virologic response = SVR. Data are shown as mean ± SD, where *, **, ***, and **** indicate P values <0.05, <0.01, <0.001, and <0.0001.

Both patients with cHCV and on-target T cells and HCV patients at the SVR stage showed a significantly higher frequency of Eomes+ TOX+ HCV-specific CD8+ T cells than patients at the chronic stage with off-target T cells and patients with rHCV (**Figure 3A**). However, patients with rHCV showed a significantly higher frequency of TOX+ T-bet+ HCV-specific CD8+ T cells (**Figure 3A**). We observed a slight increase of TOX+ CD39+ HCV-specific CD8+ T cells of aHCV and SVR (**Figure 3B**). A significant increase of CD73+Tox+ HCV-specific CD8+ T cells was observed in SVR and rHCV (**Figure 3B**). An increase in the co-expression of the immune checkpoint molecules PD-1, TIGIT, and KLRG1 on TOX+ HCV-specific CD8+ T cells was detected in patients with cHCV and on-target T cells and patients with SVR (**Figure 3C**). Interestingly, there was an increase in CD226+ TOX+ HCV-specific CD8+ T cells in aHCV, rHCV, and SVR compared to patients with cHCV with on-target T cells (**Figure 3D**).

### Patients With cHCV and Corresponding Viral Sequence Represent a Population of Terminally Exhausted CD127^-^PD1^hi^ HCV-Specific CD8+ T Cells

To determine the differentiation status of the Tex populations at different HCV stages, we analyzed the samples according to their CD45RO and CCR7 expression. Based on the differentiation markers CD45RO and CCR7, we defined naïve (CCR7+, CD45RO-), central memory (Tcm, CCR7+, CD45RO+), effector memory (Tem, CCR7-, CD45RO+), and TEM/TEMRA (Tem/Temra, CCR7-, CD45RO-) subsets of bulk CD8+ T cells and HCV-specific CD8+ T cells at different stages of infection. Representative FACS plots are shown in **Supplementary Figure S6A**. Regardless of the HCV infection status, the frequency of Tem was increased in HCV-specific CD8+ T cells compared to the bulk population (**Supplementary Figure S6B**).

**Figure 4:**
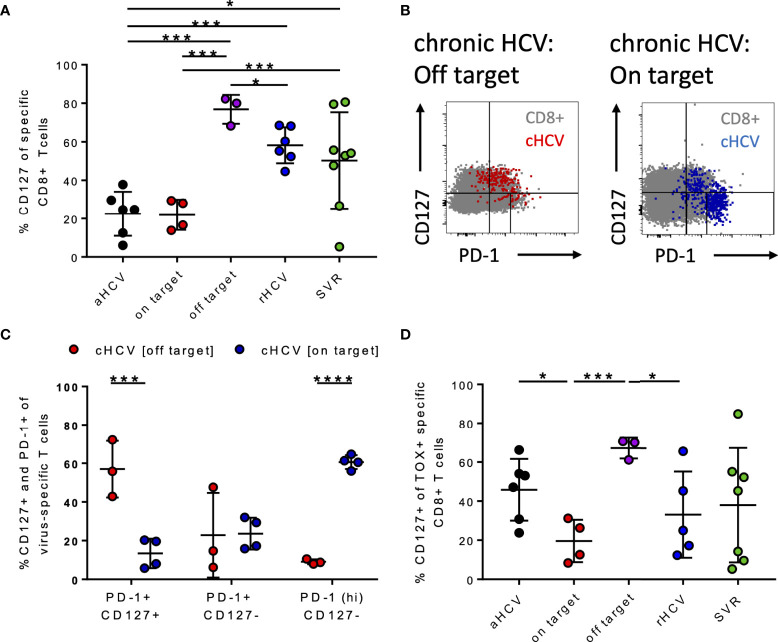
**(A–D)** Differentiation status of HCV-specific CD8+ T cells at different stages of HCV infection. **(A)** Frequency of CD127+ virus-specific CD8+ T cells at the different stages of HCV infection. **(B)** Representative dot plots for CD127 and PD-1 co-expression (gray: bulk population, red: cHCV off-target, blue: cHCV on-target). **(C)** Frequency of PD-1+ CD127+, PD1+ CD127-, and PD1^hi^ CD127- virus-specific CD8+ T cells in chronic HCV. **(D)** Frequency of CD127+ TOX+ virus-specific CD8+ T cells at all the different stages of hepatitis C infection; aHCV, acute HCV; chronic HCV with on-target T cells = on target, chronic HCV with off-target T cells = off target, rHCV, spontaneously resolved HCV; SVR, successfully treated patients with a sustained virologic response. Data are shown as mean ± SD, where *, ***, and **** indicate P values <0.05, <0.001, and <0.0001.

CD127+ HCV-specific CD8+ T cells in patients with hepatitis C infection were analyzed. Here, we detected that the patients with aHCV and cHCV and on-target T cells had a significantly lower frequency of CD127+ HCV-specific CD8+ T cells compared to patients with cHCV and off-target T cells, rHCV, and SVR (**Figure 4A**). We next investigated whether there is a correlation between the frequencies of TOX+ virus-specific CD8+ T cells and CD127+ virus-specific CD8+ T cells. Indeed, there was a negative correlation between the frequency of TOX+ and CD127+ virus-specific CD8+ T cells in chronic HCV (**Supplementary Figure S6C**). **Supplementary Figure S6C** highlights the samples with on-target T cells with red dots and patients with off-target T cells with purple dots.

Wieland et al. previously demonstrated that the appearance of memory Tex HCV-specific CD8+ T cells at the chronic stage of hepatitis C infection is defined by TCF1, PD1, and CD127 (7). **Figure 4B** shows representative dot plots for the CD127+ and PD1+ T cells of patients with cHCV and on-target T cells and off-target T cells to the circulating viral sequence. By comparing these two groups, patients with on-target T cells had significantly higher frequencies of CD127^-^PD1^hi^ HCV-specific CD8+ T cells than those with off-target T cells (**Figure 4C**). Patients with off-target T cells had significantly higher frequencies of CD127^+^PD1^+^ virus-specific CD8+ T cells compared to patients with cHCV and on-target T cells (**Figure 4C**).

These findings are in line with Wieland et al. and Kasprowicz et al., who each demonstrated that persistent antigen in the absence of escape mutations results in terminally exhausted HCV-specific CD8+ T cells in cHCV (5, 7, 8).

To assess whether TOX+ virus-specific CD8+ T cells are memory Tex, we examined the co-expression of CD127 at the different disease stages of HCV infection. Here, it was shown that there is a significant increase in TOX+ CD127+ HCV-specific CD8+ T cells at the chronic stage of infection with off-target T cells (**Figure 4D**).

### The Different Phenotypic Pattern of TOX+ Virus-Specific CD8+ T Cells Depends on the Co-Expression of T-Bet or Eomes

To determine whether the formation of the described HCV-specific CD8+ T cells is disease-specific, we additionally analyzed FLU-specific CD8+ T cells in parallel in those same patients. FLU is an example of a disease that causes an acute infection and heals spontaneously.

In a direct comparison of the diseases, we detected significantly lower frequencies of TOX+ and Eomes+ FLU-specific CD8+ T cells compared to HCV-specific CD8+ T cells, whereas an increase in T-bet+ FLU-specific CD8+ T cells was seen (**Supplementary Figure S7A**). In FLU, there was an overall lower frequency of CD39+ virus-specific CD8+ T cells (**Supplementary Figure S7B**). The highest frequencies of CD73+ virus-specific CD8+ T cells were observed in rHCV, SVR, and FLU (**Supplementary Figure S7B**). The frequencies of PD-1+ virus-specific CD8+ T cells were significantly lower among FLU patients than at any HCV stage (**Supplementary Figure S7C**). Interestingly, we saw similar low frequencies of TIGIT+ virus-specific CD8+ T cells in rHCV and FLU (**Supplementary Figure S7C**). In contrast, the frequency of KLRG1+ virus-specific CD8+ T cells among FLU patients was similar to that of cHCV patients and lowest in aHCV (**Supplementary Figure S7C**). The highest frequency of CD226+ virus-specific CD8+ T cells can be found among FLU patients. CD127+ virus-specific CD8+ T cells are also highest in FLU and rHCV (**Supplementary Figure S7D**).

**Figure 5:**
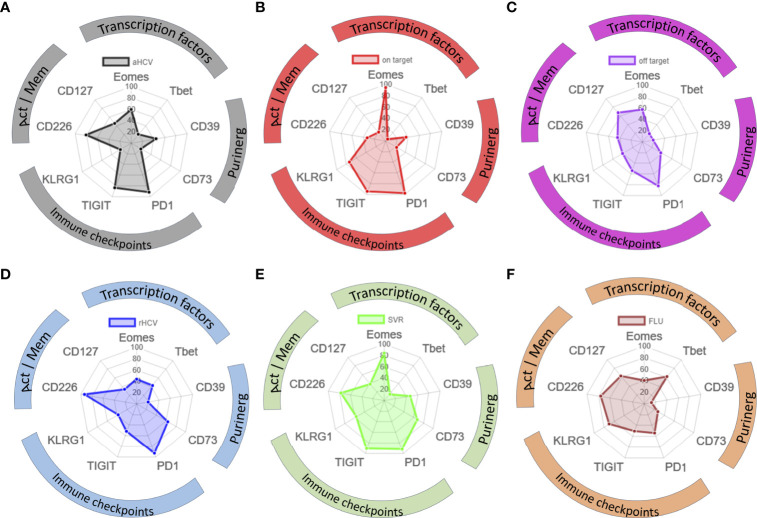
**(A–F)** Phenotypic pattern of TOX+ HCV-specific and TOX+ FLU-specific CD8+ T cells. **(A–F)** Phenotypic pattern of HCV-specific and FLU-specific CD8+ T cells in radar plots: Act, activation; Mem, memory; aHCV, acute HCV; on target = chronic HCV with on-target T cells, off target = chronic HCV with off-target T cells, rHCV, spontaneously resolved HCV; SVR, successfully treated patients with a sustained virologic response; FLU, influenza.

To compare the phenotypic pattern of TOX+ HCV-specific CD8+ T cells at the different disease stages of HCV as well as of FLU-specific T cells, we created radar plots (**Figures 5A** **–F**). Here, we discovered that the expression pattern of the indicated markers among TOX+ virus-specific CD8+ T cells differed depending on the disease stage of the HCV infection, or FLU-specific CD8+ T cells as an internal comparison. Overall, there were similarities between patients with cHCV with on-target T cells and HCV patients with SVR defined by a high presence of Eomes and increased expression of immune checkpoint molecules (PD1, TIGIT, and KLRG1) (**Figures 5B, E** **)**. Nevertheless, SVR showed higher frequencies of CD226+ TOX+ HCV-specific CD8+ T cells and CD73+ TOX+ virus-specific CD8+ T cells (**Figure 5E**). In contrast, phenotypic similarities between cHCV patients with off-target T cells and rHCV were characterized by an increase in CD73+ TOX+ virus-specific CD8+ T cells and CD127+ TOX+ HCV-specific CD8+ T cells (**Figures 5C, D** **)**. The TOX+ HCV-specific CD8+ T cells at the acute HCV stage showed an increased appearance of CD39+ TOX+ virus-specific CD8+ T cells and CD226+ TOX+ virus-specific CD8+ T cells (**Figure 5A**). In FLU-specific T cells, TOX+ virus-specific CD8+ T cells were characterized by a high frequency of T-bet+ TOX+ virus-specific CD8+ T cells and a much lower co-expression of the immune checkpoint molecules PD-1, TIGIT, and KLRG1 (**Figure 5F**).

**Figure 6:**
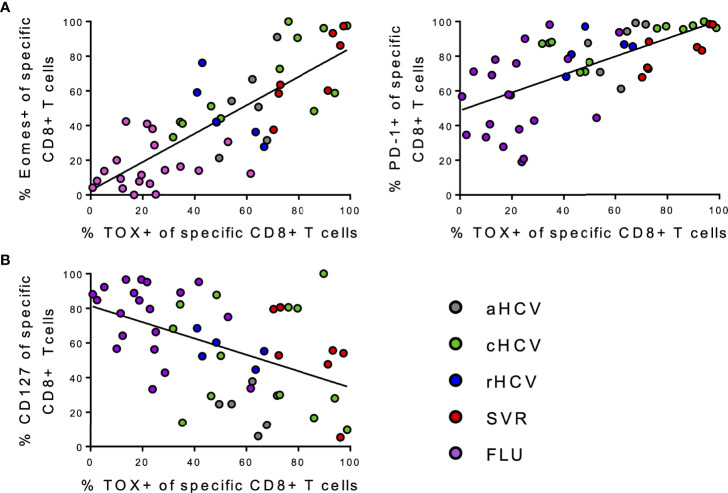
**(A, B)** Correlation analysis of the indicated markers regarding all individual virus specific CD8+ T cells samples included in the study. **(A)** Correlations between frequencies of Eomes+, PD-1+ virus-specific CD8+ T cells, and frequencies of TOX+ virus-specific CD8+ T cells. **(B)** Correlations between frequencies of CD127+ virus-specific CD8+ T cells and frequencies of TOX+ virus-specific CD8+ T cells.

Interestingly, comparing the expression at all disease stages and additionally of FLU specific CD8+ T cells, the frequencies of both Eomes+ virus-specific CD8+ T cells and PD1+ virus-specific CD8+ T cells were found to be positively correlated with TOX+ virus-specific CD8+ T cells (**Figure 6A**). Whereas TOX+ virus-specific CD8+ T cells negatively correlated with CD127+ virus-specific CD8+ T cells (**Figure 6B**). Correlations of frequencies at TOX+ virus-specific CD8+ T cells with the remaining markers in the panel are shown in **Supplementary Figure S8**.

## Discussion

Here, we analyzed the immune phenotypic pattern of HCV-specific CD8+ T cells at different stages of hepatitis C infection. We observed that TOX is expressed in HCV-specific CD8+ T cells to a certain degree at each stage of hepatitis C infection. Several studies have already shown that TOX expression occurs in the different Tex subsets (Progenitor Tex, Intermediate Tex, and Terminal Tex) (28, 29, 56–58) and that further differentiation is modulated by the interaction of additional transcription factors TCF1, T-bet, Eomes, and NR4A (7, 19, 20, 57, 59, 60).

We found that TOX+ HCV-specific CD8+ T cells considerably differ between different stages of infection with hepatitis C. Thereby, the presence of mutations of the viral sequence in cHCV causes off-target T cells that manifests a different transcriptional network in the virus-specific CD8+ T cells compared to circulating autologous viral sequence. Previously, Wieland et al. and Kasprowicz et al. showed that the occurrence of off-target mutations in HCV-specific CD8+ T cells is associated with the emergence of progenitor memory Tex cells (7, 8). These progenitor exhausted T cells have previously also been described in other diseases and the LCMV mouse model (7, 8, 61). Here, the cells are characterized by upregulation of TCF1 and CD127 (7, 8, 62). In our study, TOX+ HCV-specific CD8+ T cells from patients with cHCV and off-target T cells were shown to have significantly lower Eomes expression while exhibiting a CD127^+^PD1^+^ phenotype, indicating a progenitor memory phenotype.

In contrast, we found that patients with cHCV and on-target T cells had significantly higher frequencies of TOX+ virus-specific CD8+ T cells characterized by strong Eomes expression and a CD127^-^PD1^hi^ phenotype, thus identifying them as terminally exhausted T cells. It has already been shown that terminally exhausted virus-specific CD8+ T cells exhibit a dysfunctional character characterized by reduced cytokine production and are unable to proliferate even under PD-1 blockade (7, 23, 27, 33, 63).

In contrast, TOX+ virus-specific CD8+ T cells from rHCV patients were characterized by co-expression of T-bet, CD127, and CD226. A phenotype has been described in the literature as intermediate Tex cell (26, 64). However, it is not yet clear whether the intermediate Tex cells differentiate into terminally exhausted T cells or persist as intermediate Tex cells.

In the formation of CD8+ T cell exhaustion, not only changes in expression at immune checkpoints and differentiation occur but also metabolic effects are observed (65). Thus, purinergic signaling is also affected in the context of T cell exhaustion, especially in oncological diseases, for example, gastric cancer or colorectal cancer (43, 66–68). Also in HIV infection, the role of the ectonucleotidases CD39 and CD73 has been investigated (42). High extracellular ATP levels create a pro-inflammatory environment (69). The ectonucleotidases CD39 and CD73 are involved in shaping an equilibrium of ATP and adenosine (69). Shi et al. showed that high ATP levels induced apoptosis and dysfunction of CD8+ T cells (66). Gupta et al. already showed that CD39 is a marker for terminally exhausted CD8+ virus-specific T cells in HCV and HIV (63). We were also able to detect a slight increase of CD39+ virus-specific CD8+ T cells in patients with cHCV and on-target T cells compared to cHCV patients with off-target T cells.

However, in rHCV and SVR there was a significant increase in CD73+TOX+ virus-specific CD8+ T cells compared to cHCV with on-target T cells and aHCV. To what extent the increase in CD73+ virus-specific CD8+ T cells correlates with functional relevant differences of the adenosine pathway needs to be investigated in further studies. Since CD73 also plays a role in T cell differentiation, the increase in the frequency of CD73+ virus-specific CD8+ T cells in rHCV and SVR may also be associated with differentiation into memory CD8+ T cells (42, 43).

To further evaluate whether the combination of expression of different transcription factors and immune checkpoints detected at different stages of hepatitis C infection is either disease-specific or rather a general response pattern of virus-specific CD8+ T cells to antigen presentation, we examined FLU-specific CD8+ T cells as a near-ideal control in parallel in the same patient cohort side by side. FLU represents a disease model with an acute phase, in which healing occurs over the course without persistent antigen. Interestingly, FLU-specific CD8+ T cells showed a significantly lower expression of the transcription factor TOX (**Supplementary Figure S5A**). **Figure 5F** shows that the TOX+ FLU-specific CD8+ T cells are characterized by co-expression of T-bet and CD127.

The current study has the particular strength of looking at the expression of a broad range of intra- and extracellular molecules using different HCV-specific tetramers specific for different HCV epitopes, for which the corresponding circulating virus sequence was established, and known to be restricted by different HLA molecules, at different stages of HCV infection including patients with acute HCV and after spontaneous resolution.

The parallel analysis of FLU-specific tetramer-positive CD8+ T cells renders the ideal internal control and comparator. However, a direct comparison between the HCV-specific CD8 + T cells and the FLU-specific CD8+ T cells was not possible by tSNE analyses by using two separate panels (**Supplementary Figure S2**). While the functionality of the HCV T cells with high expression of exhaustion markers has not been tested in this study there is ample literature directly linking PD-1 expression with disturbed functionality (18, 27, 33, 70). Further experiments should investigate the functionality of Tex cells and further investigate metabolic changes in purinergic signaling. Other limitations of this study include a small sample size and lacking longitudinal data with standardized sampling.

Future prospective studies should look at patients with acute infection with known natural disease course, or chronic patients before, during, and at defined time points after achieving SVR – with defined DAA therapies. Additional data of the intrahepatic HCV-specific CD8+ T cell response, for example, using liver aspiration techniques will surely render further data.

Overall, the current data rather shows that on the one hand there were distinct transcriptional and phenotypic fingerprints observed for HCV-specific T cells at different disease stages and that the exhaustion pattern of virus-specific CD8+ T cells persisted after reaching SVR in hepatitis C virus infection. There was a seemingly direct correlation of expression of TOX and Eomes with the expression of exhaustion molecules in HCV as well as FLU-specific T cells. With a lesser degree of immune exhaustion detected in T cells specific for escaped epitopes, in patients with spontaneously resolved infection as well as FLU specific epitopes questions of the reversibility and a hypothetical point of no return – once a transcriptional factor program for exhaustion is detectable in a virus-specific T cell (7, 71).

Our results support the hypothesis that TOX can be detected in different Tex cell subsets (28, 29, 56), and that TOX influences the fate of the T cells together with additional transcription factors. In addition, the purinergic signaling pathway *via* CD39 and CD73 may turn out to be a system that can be influenced in the context of T cell exhaustion. In terms of avenues for potential future clinical translation, TOX should be tested as a new target for immunotherapy in cancer and infectious diseases to increase T cell functionality and counteract the dysfunctional immune response of CD8+ T cells.

## Data Availability Statement

The datasets presented in this study can be found in online repositories. The names of the repository/repositories and accession number(s) can be found in the article/**Supplementary Material**.

## Ethics Statement

The studies involving human participants were reviewed and approved by the Ärztekammer Hamburg WF14-09, PV4780, PV4081. The patients/participants provided their written informed consent to participate in this study.

## Author Contributions

NW and JS designed the study. NW, FB, and JS designed the panels. NW, AW, and VD conducted experiments. NW analyzed the data. NW, CB, and JS recruited the patients. JS supervised the study at all stages. NW and JS wrote the first draft. SP, SH, FH, and JT gave institutional support. All authors reviewed the manuscript and gave important input.

## Funding

This project has been funded by the Deutsche Forschungsgemeinschaft SFB841 (NW, SH, and JS) and SFB1328 (SH, FH and JS), and Deutsches Zentrum für Infektionsforschung DZIF (SH, CB, and JS). The funders had no role in study design, data collection, analysis, decision to publish, or preparation of the manuscript.

## Conflict of Interest

The authors declare that the research was conducted in the absence of any commercial or financial relationships that could be construed as a potential conflict of interest.

## Publisher’s Note

All claims expressed in this article are solely those of the authors and do not necessarily represent those of their affiliated organizations, or those of the publisher, the editors and the reviewers. Any product that may be evaluated in this article, or claim that may be made by its manufacturer, is not guaranteed or endorsed by the publisher.
