# Supplementary material for: Transcriptional Pattern Analysis of Virus-Specific CD8+ T Cells in Hepatitis C Infection: Increased Expression of TOX and Eomesodermin During and After Persistent Antigen Recognition
Source: Front Immunol. 2022 Jun 6;13:886646. doi: 10.3389/fimmu.2022.886646 (PMC9207347; doi:10.3389/fimmu.2022.886646)
Supplement: Supplementary file 1 [file Presentation_1.pptx]

## Slide 1
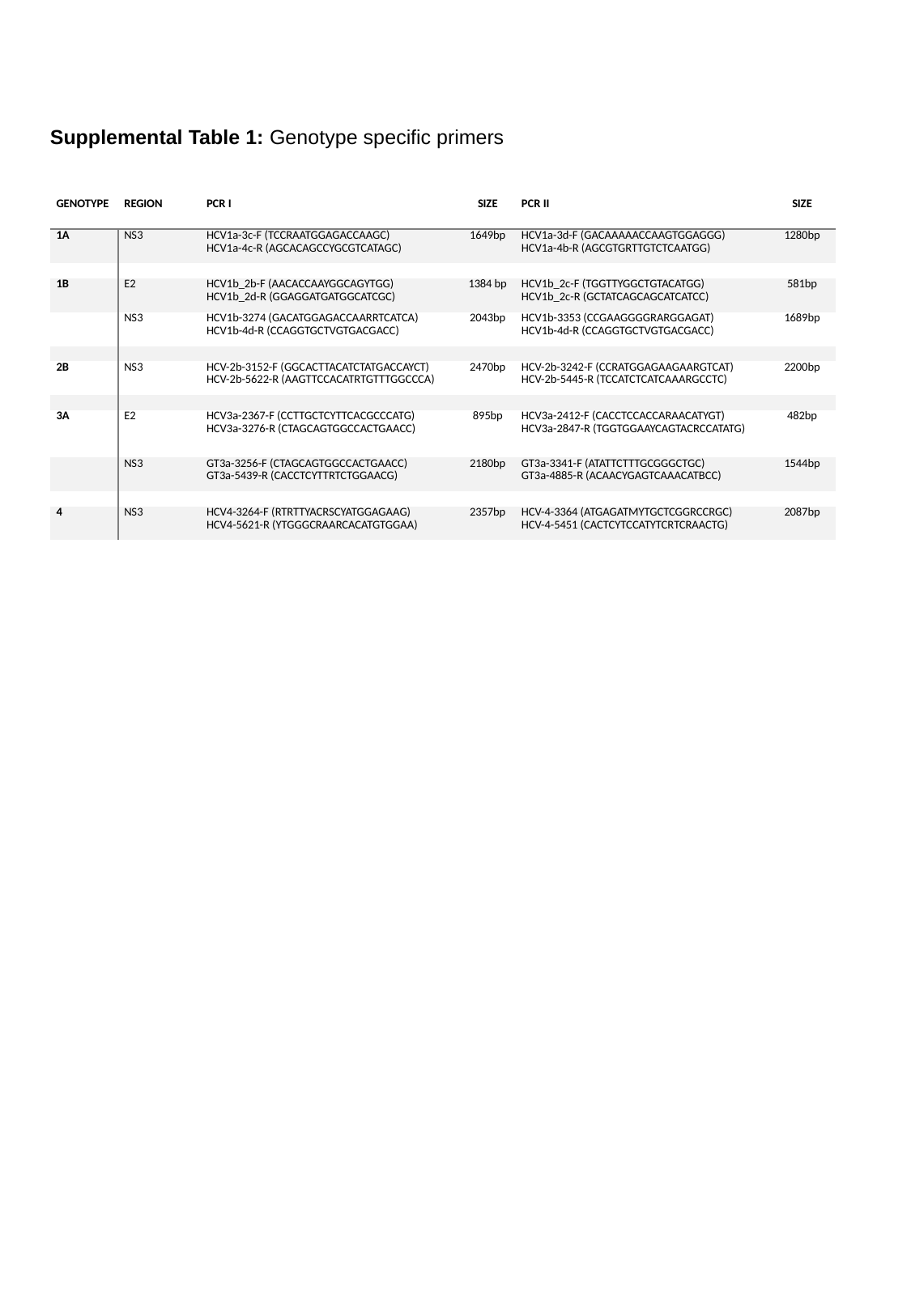

Supplemental Table 1: Genotype specific primers
| Genotype | Region | PCR I | Size | PCR II | Size |
| --- | --- | --- | --- | --- | --- |
| 1a | NS3 | HCV1a-3c-F (TCCRAATGGAGACCAAGC) HCV1a-4c-R (AGCACAGCCYGCGTCATAGC) | 1649bp | HCV1a-3d-F (GACAAAAACCAAGTGGAGGG) HCV1a-4b-R (AGCGTGRTTGTCTCAATGG) | 1280bp |
| | | | | | |
| 1b | E2 | HCV1b\_2b-F (AACACCAAYGGCAGYTGG) HCV1b\_2d-R (GGAGGATGATGGCATCGC) | 1384 bp | HCV1b\_2c-F (TGGTTYGGCTGTACATGG) HCV1b\_2c-R (GCTATCAGCAGCATCATCC) | 581bp |
| | NS3 | HCV1b-3274 (GACATGGAGACCAARRTCATCA) HCV1b-4d-R (CCAGGTGCTVGTGACGACC) | 2043bp | HCV1b-3353 (CCGAAGGGGRARGGAGAT) HCV1b-4d-R (CCAGGTGCTVGTGACGACC) | 1689bp |
| | | | | | |
| 2b | NS3 | HCV-2b-3152-F (GGCACTTACATCTATGACCAYCT) HCV-2b-5622-R (AAGTTCCACATRTGTTTGGCCCA) | 2470bp | HCV-2b-3242-F (CCRATGGAGAAGAARGTCAT) HCV-2b-5445-R (TCCATCTCATCAAARGCCTC) | 2200bp |
| | | | | | |
| 3a | E2 | HCV3a-2367-F (CCTTGCTCYTTCACGCCCATG) HCV3a-3276-R (CTAGCAGTGGCCACTGAACC) | 895bp | HCV3a-2412-F (CACCTCCACCARAACATYGT) HCV3a-2847-R (TGGTGGAAYCAGTACRCCATATG) | 482bp |
| | NS3 | GT3a-3256-F (CTAGCAGTGGCCACTGAACC) GT3a-5439-R (CACCTCYTTRTCTGGAACG) | 2180bp | GT3a-3341-F (ATATTCTTTGCGGGCTGC) GT3a-4885-R (ACAACYGAGTCAAACATBCC) | 1544bp |
| | | | | | |
| 4 | NS3 | HCV4-3264-F (RTRTTYACRSCYATGGAGAAG) HCV4-5621-R (YTGGGCRAARCACATGTGGAA) | 2357bp | HCV-4-3364 (ATGAGATMYTGCTCGGRCCRGC) HCV-4-5451 (CACTCYTCCATYTCRTCRAACTG) | 2087bp |

## Slide 2
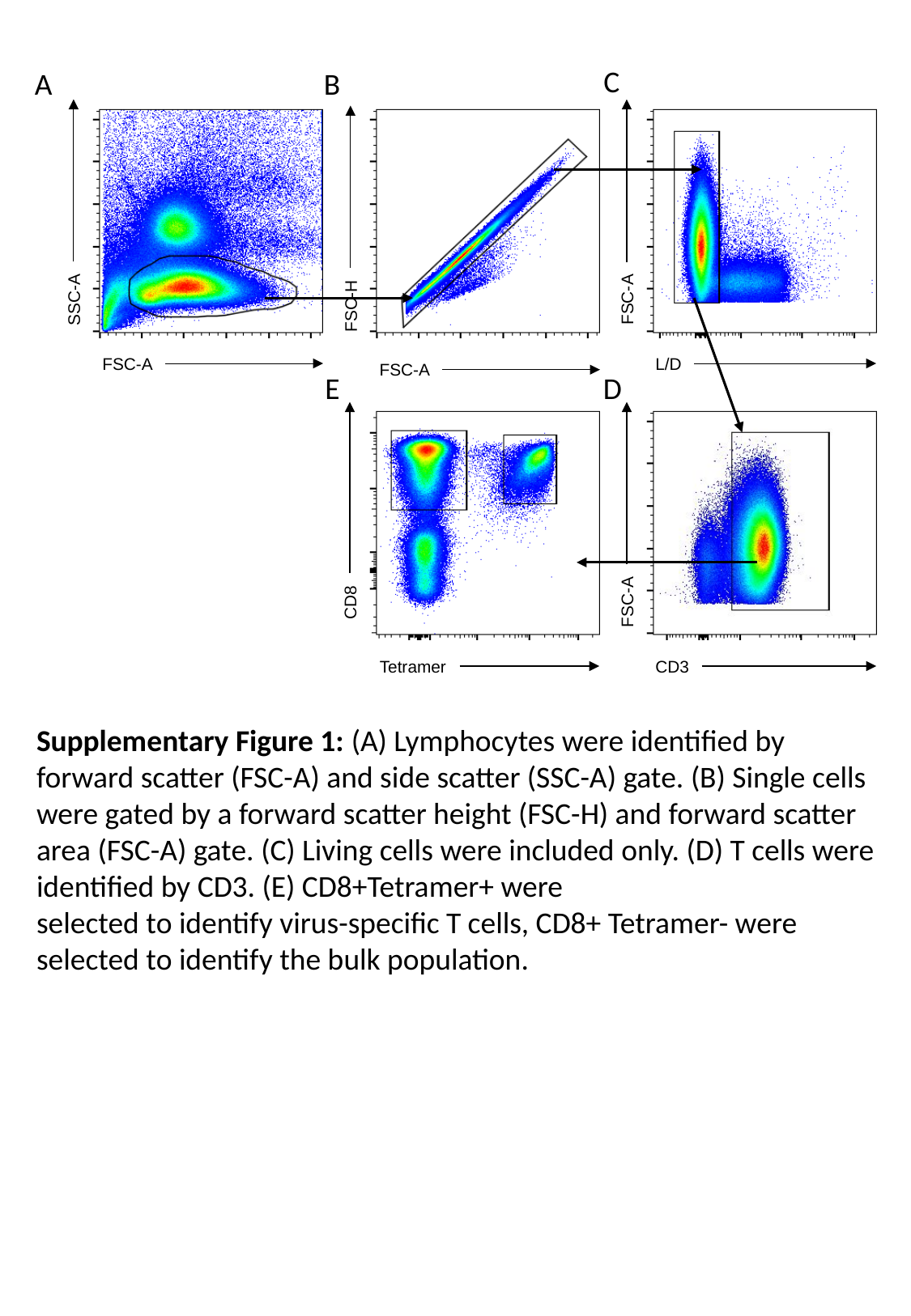

C
B
A
SSC-A
FSC-A
FSC-H
FSC-A
L/D
FSC-A
CD8
FSC-A
Tetramer
CD3
E
D
Supplementary Figure 1: (A) Lymphocytes were identified by forward scatter (FSC-A) and side scatter (SSC-A) gate. (B) Single cells were gated by a forward scatter height (FSC-H) and forward scatter area (FSC-A) gate. (C) Living cells were included only. (D) T cells were identified by CD3. (E) CD8+Tetramer+ were
selected to identify virus-specific T cells, CD8+ Tetramer- were selected to identify the bulk population.

## Slide 3
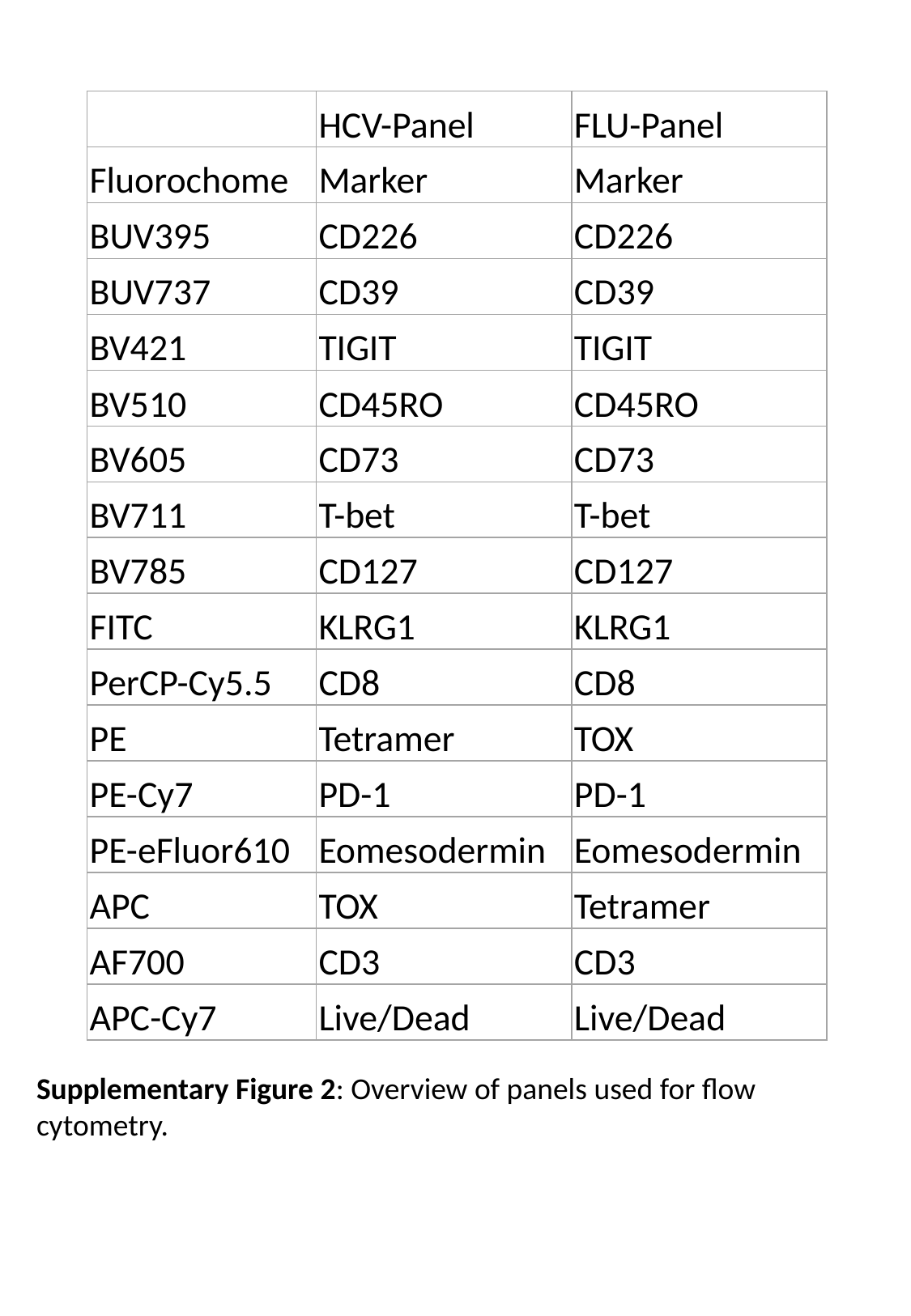

| | HCV-Panel | FLU-Panel |
| --- | --- | --- |
| Fluorochome | Marker | Marker |
| BUV395 | CD226 | CD226 |
| BUV737 | CD39 | CD39 |
| BV421 | TIGIT | TIGIT |
| BV510 | CD45RO | CD45RO |
| BV605 | CD73 | CD73 |
| BV711 | T-bet | T-bet |
| BV785 | CD127 | CD127 |
| FITC | KLRG1 | KLRG1 |
| PerCP-Cy5.5 | CD8 | CD8 |
| PE | Tetramer | TOX |
| PE-Cy7 | PD-1 | PD-1 |
| PE-eFluor610 | Eomesodermin | Eomesodermin |
| APC | TOX | Tetramer |
| AF700 | CD3 | CD3 |
| APC-Cy7 | Live/Dead | Live/Dead |
Supplementary Figure 2: Overview of panels used for flow cytometry.

## Slide 4
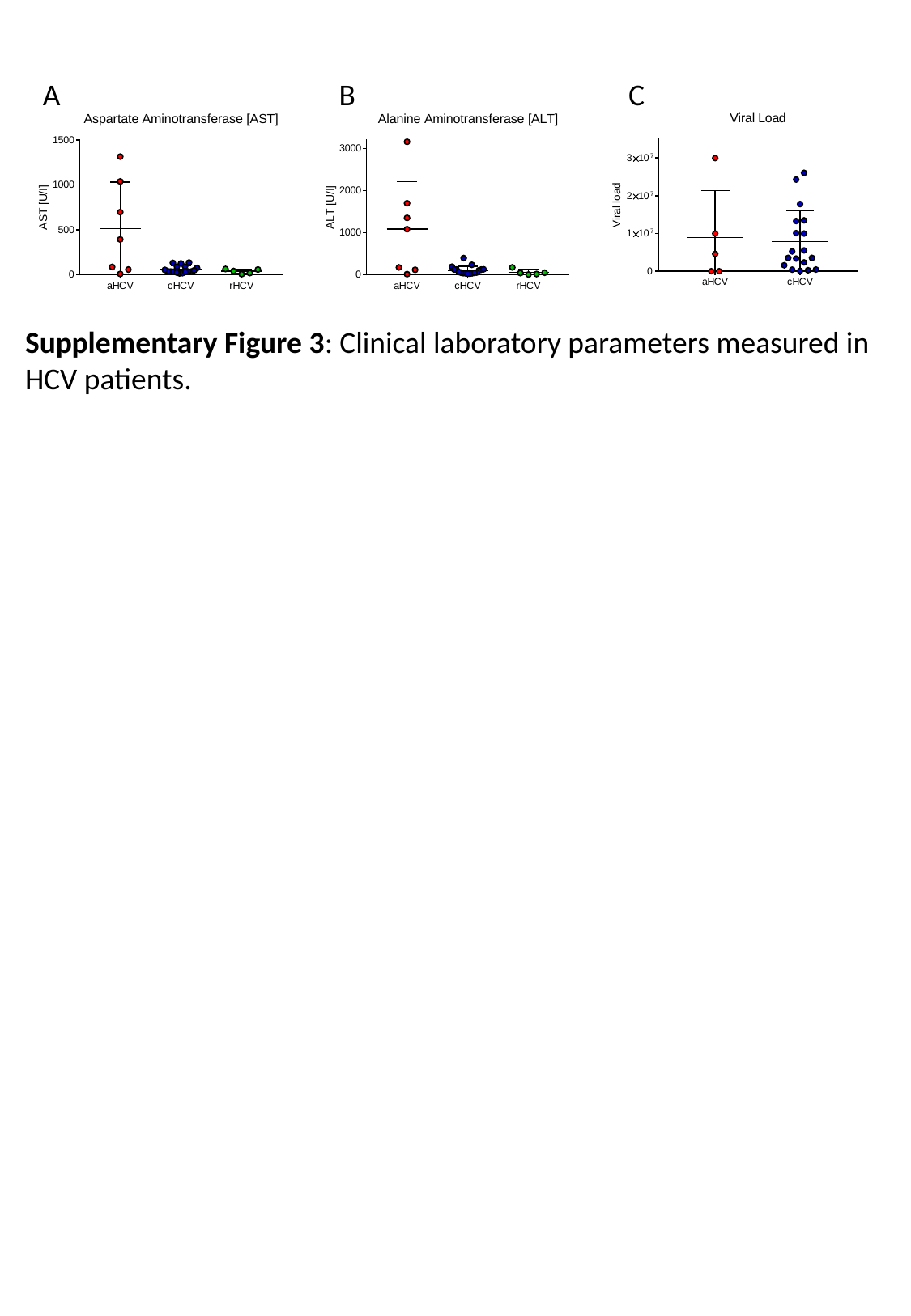

A
B
C
Supplementary Figure 3: Clinical laboratory parameters measured in HCV patients.

## Slide 5
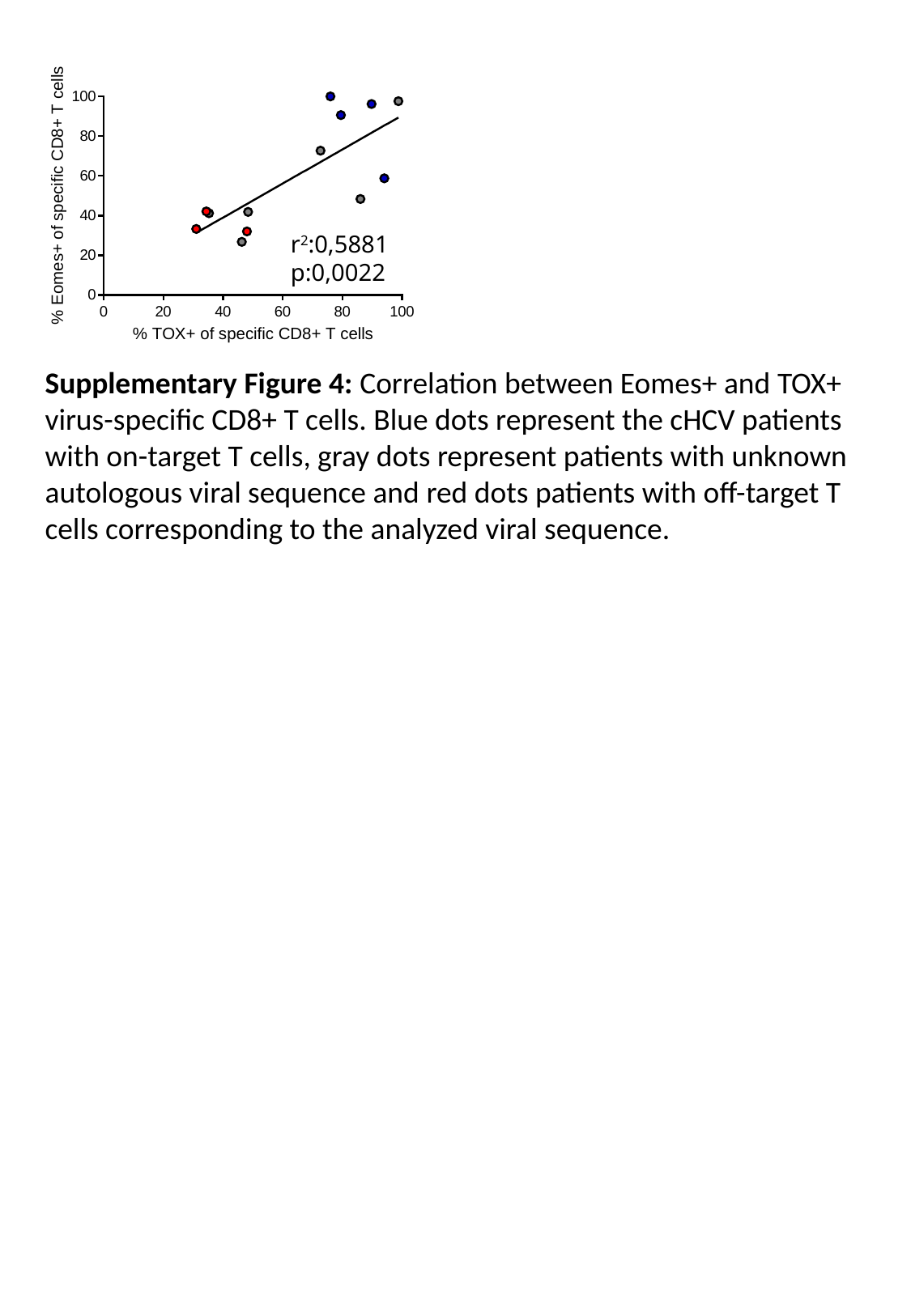

r2:0,5881
p:0,0022
Supplementary Figure 4: Correlation between Eomes+ and TOX+
virus-specific CD8+ T cells. Blue dots represent the cHCV patients with on-target T cells, gray dots represent patients with unknown autologous viral sequence and red dots patients with off-target T cells corresponding to the analyzed viral sequence.

## Slide 6
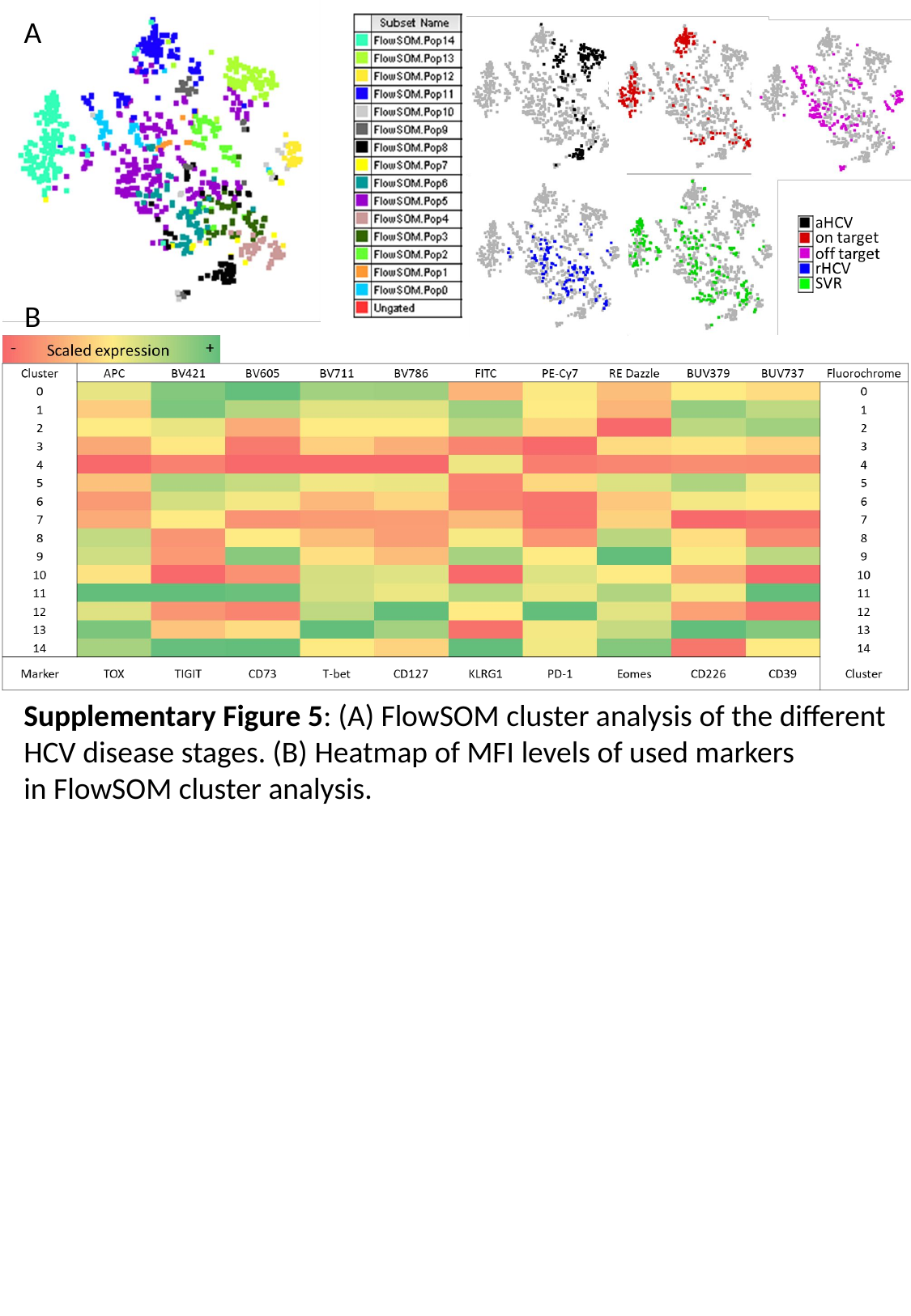

A
B
Supplementary Figure 5: (A) FlowSOM cluster analysis of the different HCV disease stages. (B) Heatmap of MFI levels of used markers in FlowSOM cluster analysis.

## Slide 7
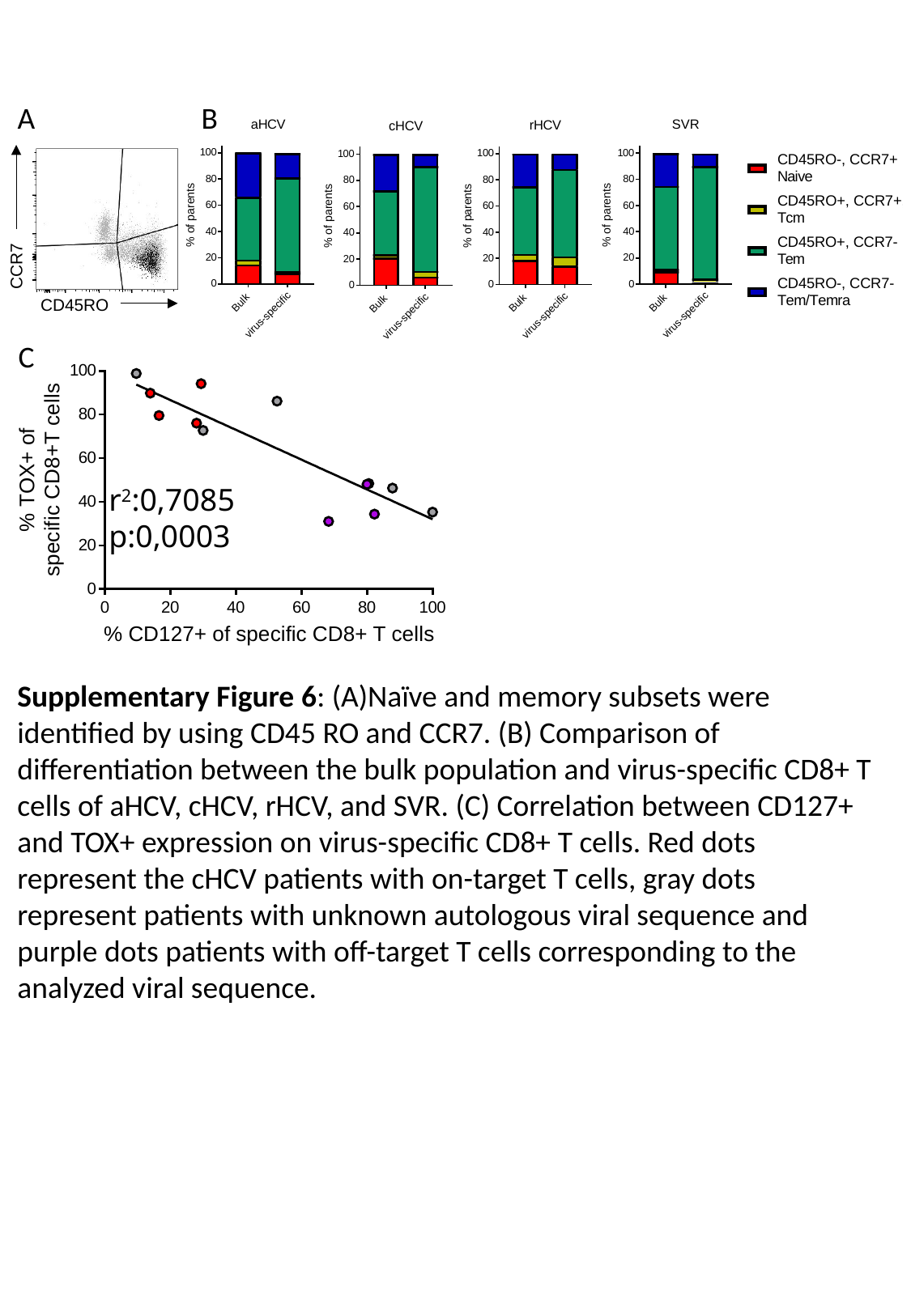

A
B
CCR7
CD45RO
C
r2:0,7085
p:0,0003
Supplementary Figure 6: (A)Naïve and memory subsets were identified by using CD45 RO and CCR7. (B) Comparison of differentiation between the bulk population and virus-specific CD8+ T cells of aHCV, cHCV, rHCV, and SVR. (C) Correlation between CD127+ and TOX+ expression on virus-specific CD8+ T cells. Red dots represent the cHCV patients with on-target T cells, gray dots represent patients with unknown autologous viral sequence and purple dots patients with off-target T cells corresponding to the analyzed viral sequence.

## Slide 8
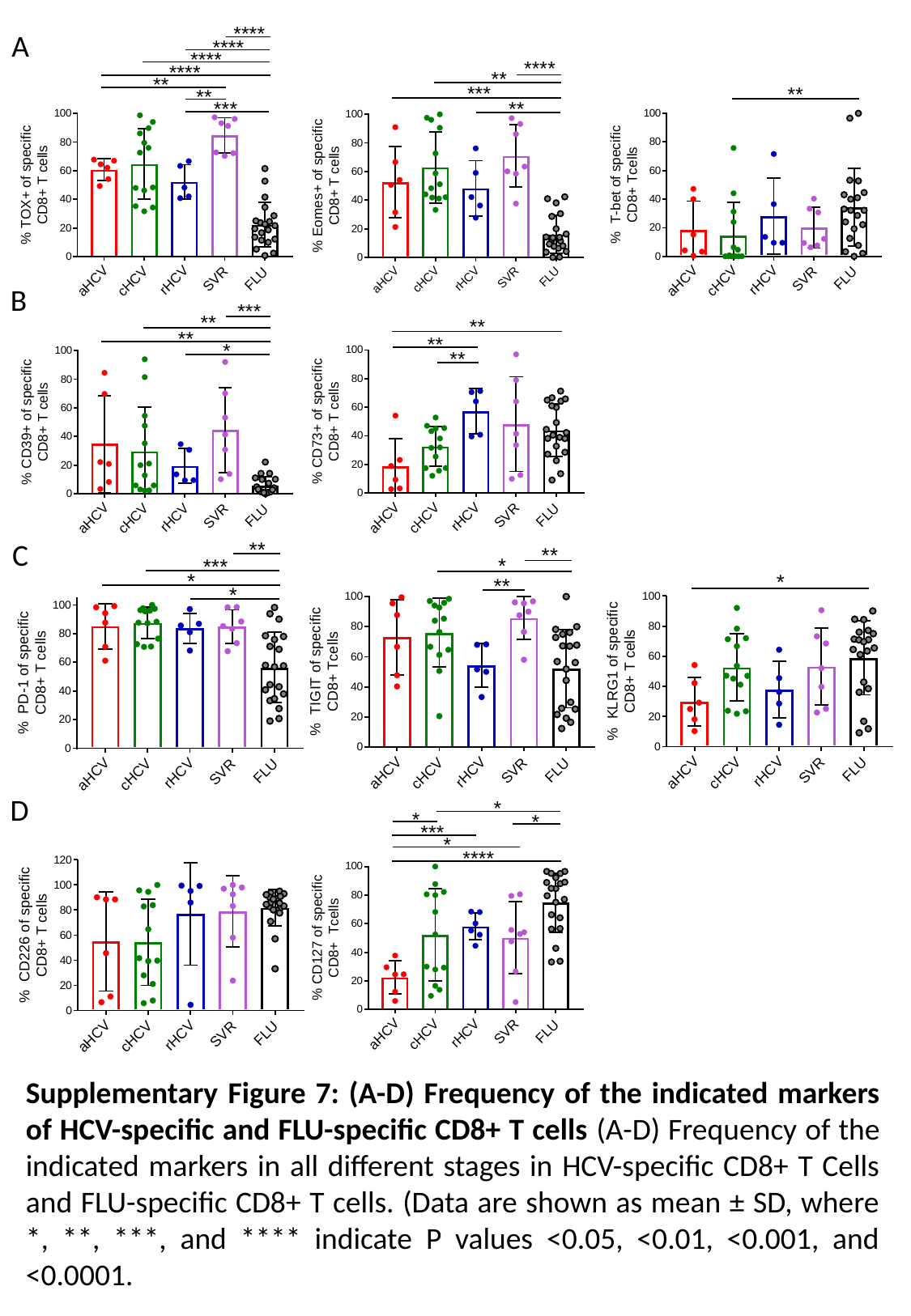

A
B
C
D
Supplementary Figure 7: (A-D) Frequency of the indicated markers of HCV-specific and FLU-specific CD8+ T cells (A-D) Frequency of the indicated markers in all different stages in HCV-specific CD8+ T Cells and FLU-specific CD8+ T cells. (Data are shown as mean ± SD, where *, **, ***, and **** indicate P values <0.05, <0.01, <0.001, and <0.0001.

## Slide 9
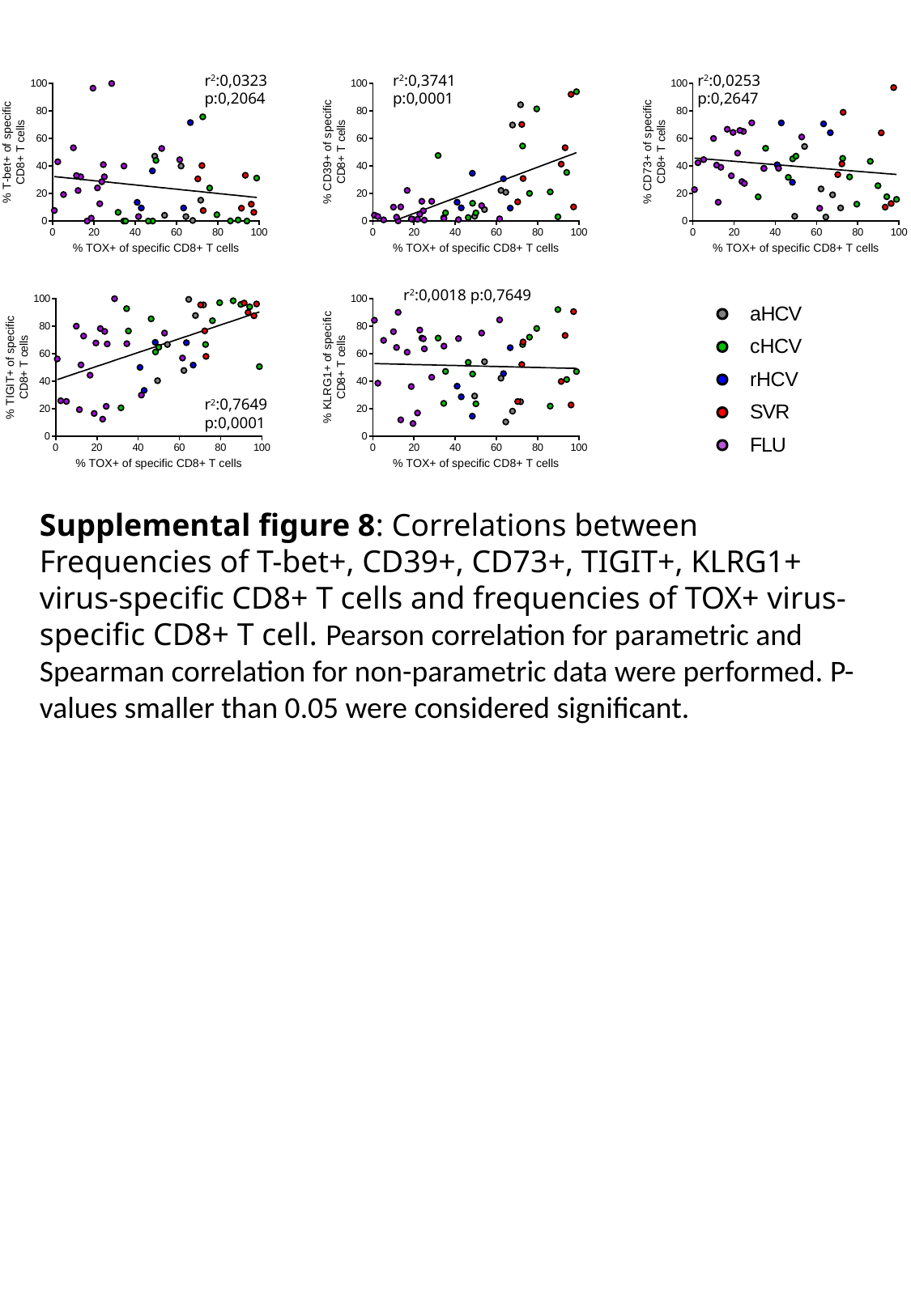

r2:0,0323
p:0,2064
r2:0,3741
p:0,0001
r2:0,0253
p:0,2647
r2:0,0018 p:0,7649
r2:0,7649
p:0,0001
Supplemental figure 8: Correlations between Frequencies of T-bet+, CD39+, CD73+, TIGIT+, KLRG1+ virus-specific CD8+ T cells and frequencies of TOX+ virus-specific CD8+ T cell. Pearson correlation for parametric and Spearman correlation for non-parametric data were performed. P-values smaller than 0.05 were considered significant.
